# MetaQuad: shared informative variants discovery in metagenomic samples

**DOI:** 10.1093/bioadv/vbae030

**Published:** 2024-02-24

**Authors:** Sheng Xu, Daniel C Morgan, Gordon Qian, Yuanhua Huang, Joshua W K Ho

**Affiliations:** School of Biomedical Sciences, Li Ka Shing Faculty of Medicine, The University of Hong Kong, Pokfulam, Hong Kong SAR, China; Laboratory of Data Discovery for Health Limited (D24H), Hong Kong Science Park, Hong Kong SAR, China; School of Biomedical Sciences, Li Ka Shing Faculty of Medicine, The University of Hong Kong, Pokfulam, Hong Kong SAR, China; Laboratory of Data Discovery for Health Limited (D24H), Hong Kong Science Park, Hong Kong SAR, China; School of Biomedical Sciences, Li Ka Shing Faculty of Medicine, The University of Hong Kong, Pokfulam, Hong Kong SAR, China; Laboratory of Data Discovery for Health Limited (D24H), Hong Kong Science Park, Hong Kong SAR, China; School of Biomedical Sciences, Li Ka Shing Faculty of Medicine, The University of Hong Kong, Pokfulam, Hong Kong SAR, China; School of Biomedical Sciences, Li Ka Shing Faculty of Medicine, The University of Hong Kong, Pokfulam, Hong Kong SAR, China; Laboratory of Data Discovery for Health Limited (D24H), Hong Kong Science Park, Hong Kong SAR, China

## Abstract

**Motivation:**

Strain-level analysis of metagenomic data has garnered significant interest in recent years. Microbial single nucleotide polymorphisms (SNPs) are genomic variants that can reflect strain-level differences within a microbial species. The diversity and emergence of SNPs in microbial genomes may reveal evolutionary history and environmental adaptation in microbial populations. However, efficient discovery of shared polymorphic variants in a large collection metagenomic samples remains a computational challenge.

**Results:**

MetaQuad utilizes a density-based clustering technique to effectively distinguish between shared variants and non-polymorphic sites using shotgun metagenomic data. Empirical comparisons with other state-of-the-art methods show that MetaQuad significantly reduces the number of false positive SNPs without greatly affecting the true positive rate. We used MetaQuad to identify antibiotic-associated variants in patients who underwent *Helicobacter pylori* eradication therapy. MetaQuad detected 7591 variants across 529 antibiotic resistance genes. The nucleotide diversity of some genes is increased 6 weeks after antibiotic treatment, potentially indicating the role of these genes in specific antibiotic treatments.

**Availability and implementation:**

MetaQuad is an open-source Python package available via https://github.com/holab-hku/MetaQuad.

## 1 Introduction

Strain-level identification is a popular modern metagenomics technique (Marx 2016). A bacterial strain is a genetic variant or a subtype within a bacterial species, playing a crucial role in bacterial adaptation and survival. These variants enable natural selection to take place, facilitating the bacteria’s ability to thrive in different environments. For example, within the gut microbiome community, the administration of antibiotics can exert a significant influence, necessitating species to adapt in order to ensure their survival (Koo *et al.* 2019). Understanding bacterial strains and their adaptations is a critical aspect of microbiota investigation (Yan *et al.* 2020). Microbes can exhibit an array of phenotypic properties with minor changes in their DNA code. Contradiction may arise in some microbiome literature, when different labs detect opposing associations with certain diseases (Marx 2016). Strain-level analysis of the microbiome composition may provide an additional layer of information that may lead to more robust and reproducible results.

Strain-level analysis is feasible with sequencing reads due to the ability to detect single nucleotide polymorphism (SNP) substitutions, which serve as reliable indicators of strain-level variations. Shotgun metagenomic sequencing possesses superior capability compared to 16S rRNA gene sequencing when it comes to identifying and detecting bacterial species that are present in lower abundance (Durazzi *et al.* 2021). This enables the detection of a larger number of SNPs. However, successfully identifying strain-level difference has long been a problem, especially for short-read sequencing data. Misalignment of short sequence reads may lead to false positive SNP calls. It can produce high-confidence SNPs, which are hard to filter out by variant calling algorithms, as they are likely to be supported by multiple reads together with high mapping quality (Day-Williams and Zeggini 2011). Sequencing error furthermore confounds detection of low-frequency SNPs. Excessive false positive SNPs may mask the effect of true SNPs, which in turn may lead to issues in downstream analyses (Ma *et al.* 2019).

SNPs with consistent allele frequency (AF) changes within a population are of key interest, as they offer insights into evolution by natural selection (Wiberg *et al.* 2017). To emphasize the impact of natural selection, we identify SNPs with noticeably distinct allele frequencies within a population and classify them as shared informative variants. Many tools have been developed to study strain-level differences, some of which are constructed especially for the shotgun metagenomic sequencing, such as metaSNV (Costea *et al.* 2017) and inStrain (Olm *et al.* 2021). However, to our knowledge, no tools have been developed to detect informative variants from a population of samples, due in part to the challenges of distinguishing shared informative variants from background noise. To address these limitations, we present a new computational tool called MetaQuad. MetaQuad can efficiently detect the shared informative variants, with high accuracy and precision.

## 2 Methods

### 2.1 Variant calling of shotgun metagenomic data

The variant calling pipeline consists of three main steps. First, following initial processing steps (e.g. removal of contaminations, low-quality reads, and duplicates), shotgun metagenomic sequencing reads are aligned to a database of microbial reference genomes using BWA-MEM (Li 2013) and subsequently filtered to remove low-quality reads. Next, the aligned reads from BAM files are piled up using cellsnp-lite (v1.2.0) (Huang and Huang 2021) to identify SNPs with minimum minor allele frequency (minMAF) of 0.02 within a population. The output data from cellsnp-lite usually contain a large number of microbial SNPs, including shared variants, sample-specific variants, and sequencing errors. Finally, MetaQuad utilizes a density-based clustering technique to identify shared informative variants, ensuring that each cluster includes a minimum number of samples. The shared informative variants can be further analyzed to calculate nucleotide diversity of genes or genomes.

### 2.2 MetaQuad model

**Figure 1.**
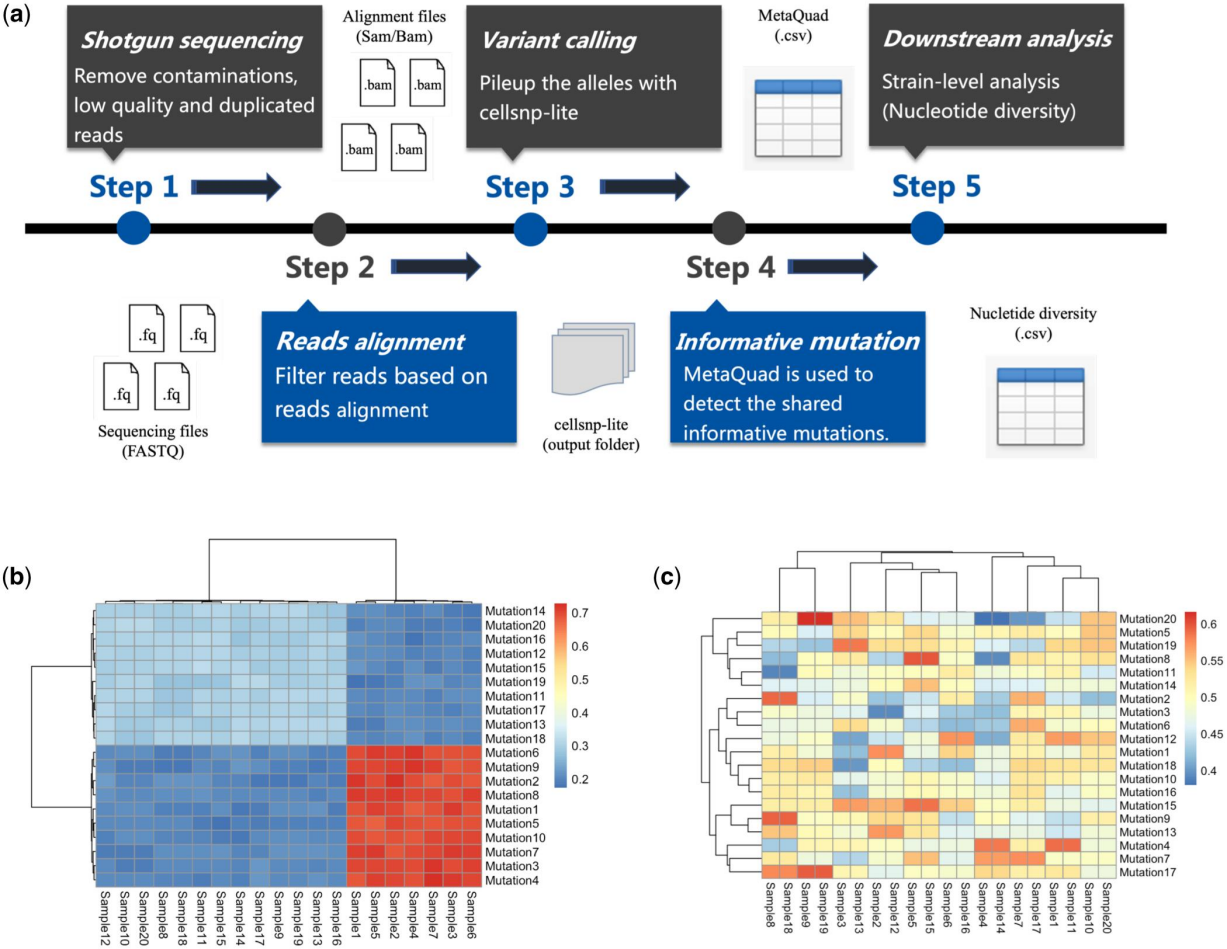
Overview of analysis pipeline and example of shared variants. (a) Recommended analysis pipeline for MetaQuad. The Shotgun metagenomic sequencing files are collected after removing contaminations (e.g. host contaminants), low-quality and duplicated reads. Filtered reads are aligned to a reference database using alignment tools, and low mapping quality reads are further filtered out. Cellsnp-lite is used to count the alleles of each sample, and the output data are processed by MetaQuad. All variants are listed in a CSV file with number of clusters, which distinguishes informative variants. Informative variants can be utilized to study the nucleotide diversity of each gene or genome. (b) Example of informative variants. Informative variants can be found in multiple samples with consistent changes in allele frequencies across populations. The allele frequencies of informative variants are similar within each population. (c) Example of random variants (background noise). Random variants can be found in one or more samples, but their allele frequencies do not have a consistent change, and the frequencies can vary greatly between samples. In the figure, different colors represent different allele frequencies.

MetaQuad is a computational method to detect shared informative variants from a population of samples. MetaQuad processes allele counts of each sample output from cellsnp-lite (Huang and Huang 2021) (Fig. 1a). The pipeline prior to MetaQuad involves cellsnp-lite calculating pileup and allele counts from bulk DNA sequencing data for each variant. MetaQuad evaluates metagenomic variants using a density-based clustering technique called OPTICS (Ordering Points To Identify Cluster Structure) (Ankerst *et al.* 1999). For each variant identified by cellsnp-lite, one-dimensional OPTICS is applied based on the AF of each sample. The AF for each variant in each sample is considered as a data point, and OPTICS clustering is employed to group similar data points. A minimal number of samples is required to form a cluster for each group.

The OPTICS algorithm consists of four main steps:

1. Reachability distance calculation: compute the reachability distance between each point and its neighboring points. The reachability distance is computed for points that are not considered core points and is utilized to determine the distance of a given point (which is not a core point) from a core point.
2. Core distance calculation: determine the core distance of each point with its nearest neighbor, based on the minimal number of samples.
3. Cluster extraction: extract clusters from regions of points that are in close proximity to each other and exhibit similar reachability distances.
4. Cluster identification: analyze the reachability distances of the points within each cluster to identify distinct groups.

MetaQuad outputs a table containing information on all detected variants from cellsnp-lite. Importantly, it calculates the estimated number of clusters for each variant. Shared informative variants can only be confirmed if there are at least two clusters present because SNPs with only one cluster may result from limitations in the reference database or sequencing errors and are therefore removed from the study. The output table also includes additional parameters, such as read depths (DPs) and allele depths (ADs), which can be helpful in filtering out false positive SNPs. The examples of informative variant and random variants (background noise) are shown in Fig. 1b and c.

There is an important parameter in MetaQuad called minSample, which refers to the minimum number of samples required in each cluster. This parameter plays a crucial role in the model as having an insufficient sample size can result in noise being incorrectly assigned to multiple clusters. In our simulation experiments, we investigated the impact of different minSample values and observed that increasing this value may decrease both the true positive and false positive rates. To strike a balance between minimizing false positives and maintaining a reasonable true positive rate, we suggest setting the minSample value to 5% of the total sample size.

### 2.3 Validity assessment of MetaQuad assumptions

MetaQuad operates under the assumption that the allele frequencies of shared informative variants are similar within a population of the human gut microbiome. In order to test the assumption, we analyzed differences across individuals using a dataset from the Broad Institute-OpenBiome Microbiome Library (BIO-ML) (Poyet *et al.* 2019), under BioProject PRJNA544527. We selected 30 shotgun metagenomic samples from three healthy individuals with different time points (10 samples each, with individuals coded as “am,” “an,” and “ao”). As certain strains should be shared between samples from the same person, so too should the variants.

We processed the data by first trimming adaptors and removing phix contamination with BBMap/bbduk.sh. To remove human contamination, we used BBMap/bbmap.sh with a minimum alignment identity parameter of 0.95, and we removed duplicated reads with PRINSEQ++ (Cantu *et al.* 2019). After these steps, we aligned the filtered reads to a reference database with BWA-MEM. Specifically, we used the coding sequences of the integrated gene catalog (IGC) (Li *et al.* 2014) database from the Human Microbiome Project as our reference. We further filtered the resulting BAM files with Samtools (Danecek *et al.* 2021), removing alternative and supplemental alignments, as well as reads with mapping quality scores ≤30 to ensure accuracy.

Given the large number of genes in the reference database, we selected only the genes with genus annotations and sample occurrence frequencies larger than 0.95 according to the IGC annotation table. After this filtering step, we identified 5160 genes and used them to study shared variants both within and across individuals.

### 2.4 Evaluating the model performance with simulated data

**Figure 2.**
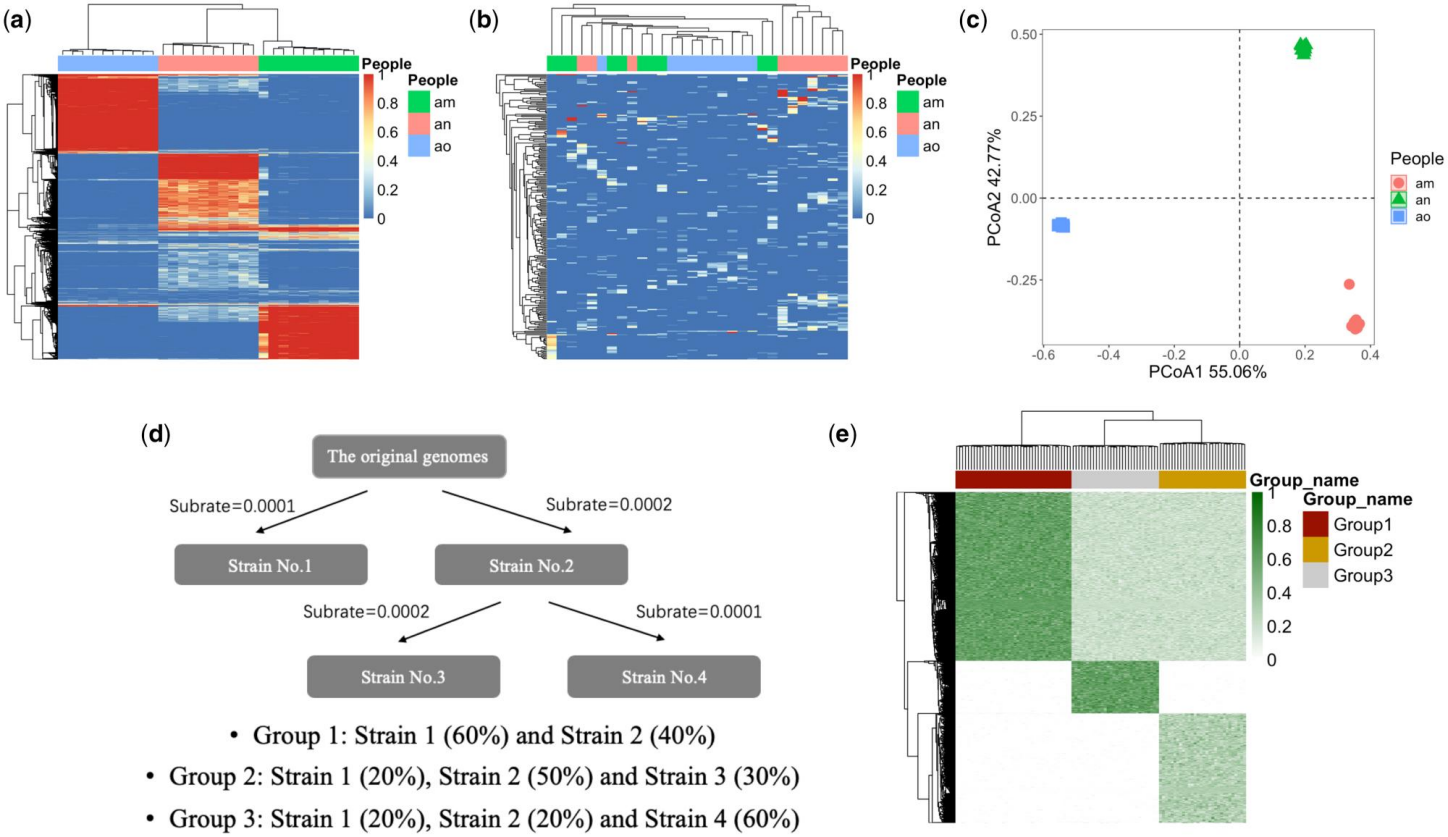
Shared informative variants in real and simulated datasets. (a) Allele frequencies of informative variants detected by MetaQuad within each individual. Colors indicate varying allele frequencies. (b) Allele frequencies of uninformative variants within each individual. Colors denote distinct allele frequencies. (c) PCoA plot of informative variants detected by MetaQuad for each individual. (d) Schematic representation of the strain simulation pipeline for the simulated dataset. (e) Allele frequencies of simulated informative variants.

Paired-end reads with a length of 151 bp were generated using InSilicoSeq (Gourlé *et al.* 2019) to simulate shotgun metagenomic sequencing with sequencing errors (error model: NovaSeq). The simulation process involved several steps. Initially, a small gut microbiome consisting of five species from the genus Fusobacterium was established. This microbiome was used as the reference genome for the simulation. The genus Fusobacterium is known to be present in the human gut, with some members promoting the development of colorectal cancer (Bi *et al.* 2022). To generate the reference genome, five Fusobacterium genomes were downloaded from NCBI (https://www.ncbi.nlm.nih.gov) with the NCBI Reference Sequence: NZ_LN831027.1 *Fusobacterium nucleatum*, NZ_JADRGD010000001.1 *Fusobacterium necrophorum*, NZ_CAEUHP010000001.1 *Fusobacterium mortiferum*, NZ_CP028103.1 *Fusobacterium varium*, and NZ_CP028105.1 *Fusobacterium ulcerans*. Four strains were generated based on the original genome, with different substitution rates (Fig. 2d). The genome variants were performed using mutate.sh from BBmap (Bushnell 2014).

Using the genomes, the simulation was performed on datasets consisting of 100 samples divided into three groups with different percentages of strains (Fig. 2d). The number of simulated informative variants was 7638, and each dataset contained 100 samples divided into three groups (Groups 1, 2, and 3). The detailed simulation process is described in Supplementary Fig. S1. In order to better detect the performance of MetaQuad, multiple datasets were simulated with different mean depths of the variants. The mean depths varied from 3 to 35.5, and the detailed information of each dataset is shown in Supplementary Table S1.

The simulated reads were trimmed using BBMap/bbduk.sh to remove bases with quality scores below 10. The reads were then aligned back to the reference genome using BWA-MEM. Variant calling was performed using different tools with their default parameters, and their performances were compared later. These tools included BCFtools (Danecek *et al.* 2021), GATK’s HaplotypeCaller (Poplin *et al.* 2017), VarScan2 (Koboldt *et al.* 2012), MetaSNV (Costea *et al.* 2017), and InStrain (Olm *et al.* 2021). The performance of these tools was evaluated using *F*1 score, true positive rate, and false positive.

### 2.5 Identification of shared variants after antibiotic treatment

Data from a study on *Helicobacter pylori* infected patients with antibiotic therapy (Wang *et al.* 2022) were used as a biological benchmark [antibiotic resistance gene (ARG) dataset]. A total of 121 stool samples were collected and subjected to metagenomic shotgun sequencing. These samples were obtained from 44 patients at three specific time points: before antibiotic treatment, 6 weeks after antibiotic treatment, and 6 months after antibiotic treatment. The data were processed the same way as before (30 samples from 3 individuals).

To identify genes relevant to antibiotic resistance, we aligned the protein sequences of the IGC database to the Comprehensive Antibiotic Resistance Database (Alcock *et al.* 2019) using DIAMOND (Buchfink *et al.* 2021). We selected genes that aligned to the CARD database with an *e*-value <e-50, resulting in 4948 ARGs. We then filtered the bam files to include only reads from these ARGs, resulting in final ARG bam files that were used for further analysis, including variant calling with different methods.

We used cellsnp-lite with a minMAF of 0.02 and minCOUNT of 100, and MetaQuad with a minSample of 6, mean AD of 0.1, and mean DP of 0.5 for variant calling. All other variant calling tools were applied with their default parameters. Additional details of the data processing can be found in Fig. 4a.

### 2.6 Runtime analysis of variant calling tools

We compared the computational efficiency of vancan2, MetaSNV, cellsnp-lite, MetaQuad, and inStrain in analyzing all samples simultaneously. For inStrain, its runtime is calculated by its own built-in equation, and the final total time is the sum of the time required for all samples. For the other tools, we recorded the times with the Unix time command (real time), using the same computational resources (ppn = 80 and storage = 500G). The CPU is an Intel(R) Xeon(R) Gold 6130 CPU @ 2.10 GHz.

### 2.7 Nucleotide diversity

Nucleotide diversity was calculated using allele frequencies of shared variants calculated by the output of MetaQuad and cellsnp-lite:

Nucleotide diversity (gene)=$\sum_{i=1}^{n}(1–\left[{{\mathrm{AF}}_{i}}^{2}+{\left(1–{\mathrm{AF}}_{i}\right)}^{2}\right])/L_{\mathrm{gene}}.$

Suppose MetaQuad detected n shared variants from a gene or a genomic region. For each shared informative variant, the AF could be calculated as the alternate allele (AD) divided by DP. The nucleotide diversity was further normalized by the length of genes (*L*_gene_). The range of nucleotide diversity was between 0 and 1, where the larger value indicated more variant accumulations.

### 2.8 Statistical methods

The OPTICS algorithm was implemented using the Python scikit-learn library (Pedregosa *et al.* 2011). Principal component analysis was performed using the prcomp function in R (version 4.0.2) and plotted using the ggbiplot function (https://github.com/vqv/ggbiplot). Multilevel pairwise comparison was performed using the pairwise.adonis function from the “pairwiseAdonis” (Martinez Arbizu 2020) package. The distance matrix was calculated using Bray–Curtis dissimilarity, and the *P*-values were adjusted using the Bonferroni method.

## 3 Results

### 3.1 Consistency of shared informative variants within each individual or group

MetaQuad was initially tested on the BIO-ML dataset, which consisted of 30 samples from 3 individuals. Since the samples were collected from healthy people, the commonalities between samples from different individuals should be much smaller than those from the same individual. When analyzed with cellsnp, 67 241 variants were detected, with a minMAF of 0.02. Out of all the variants, 64 682 had at least two clusters with a minimum sample size of 3, while the remaining variants could only be classified into a single cluster.

For MetaQuad, variants with a minimum of two clusters are considered informative. Our analysis demonstrated that the shared informative variants were highly consistent within each individual, but not across different individuals (Fig. 2a and b). Variants with only one cluster were considered likely to be noise because they were not consistent within each person (Supplementary Fig. S1). These results highlight that MetaQuad can effectively identify informative variants that distinguish between individuals (Fig. 2c).

### 3.2 MetaQuad is a robust statistical method to identify shared informative variants from shotgun metagenomic data

MetaQuad was initially benchmarked using simulated datasets, where paired-end reads were generated from InSilicoSeq (Gourlé *et al.* 2019) (Section 2). These datasets were organized into three groups, with each group containing samples that had varying percentages of strains from the original genomes (Section 2 and Fig. 2d). The allele frequencies of the simulated shared informative variants were highly consistent within each group, making the simulation more reflective of real-world scenarios. To thoroughly assess MetaQuad’s performance, multiple datasets were generated with different mean depths for the informative variants. The mean depths ranged from 3 to 35.5, and the specific details for each dataset can be found in Supplementary Table S1.

**Figure 3.**
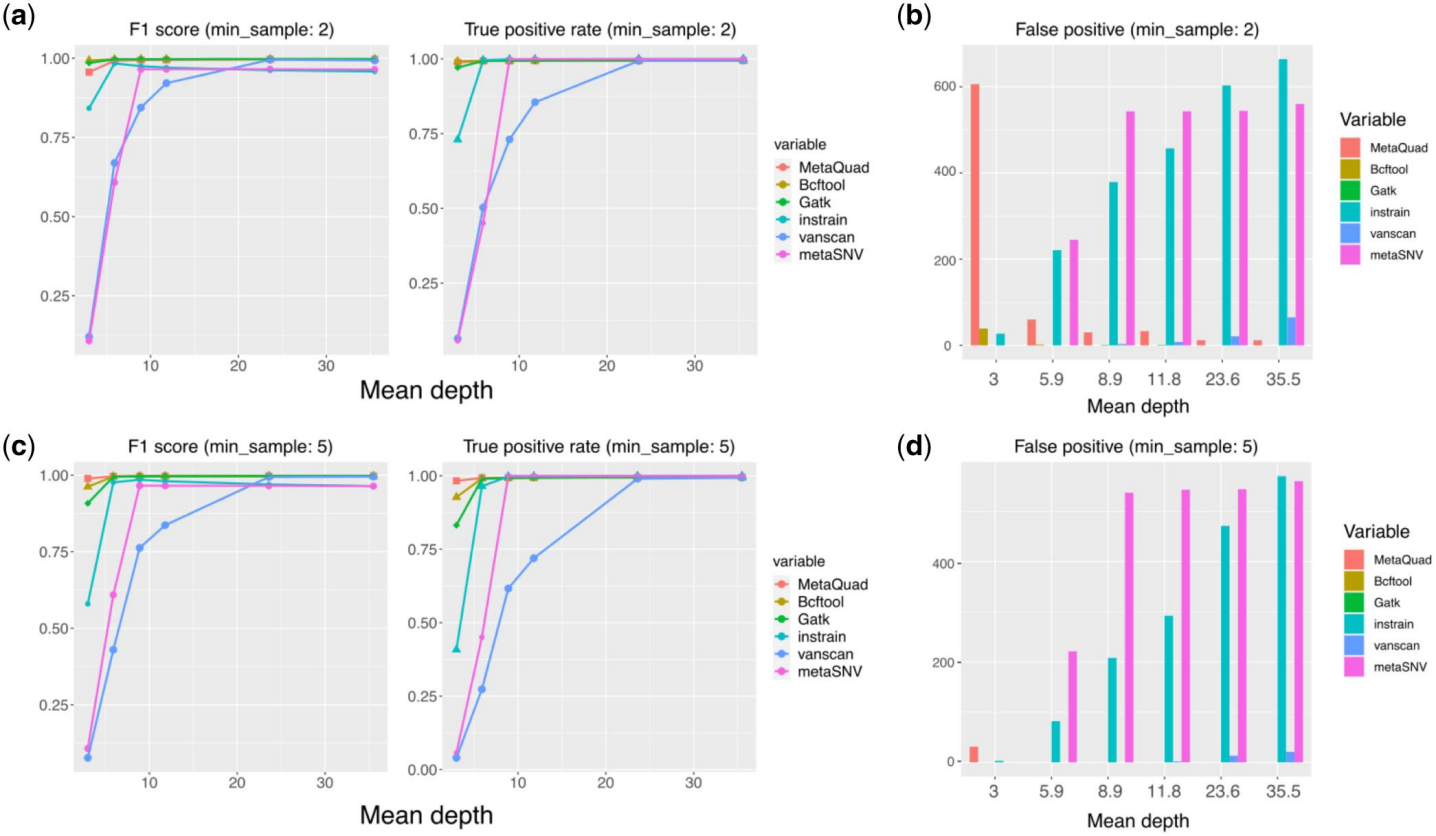
Comparison of variant calling tools. (a) *F*1 score and true positive rate of all variant calling tools, with a minimum sample threshold (min_sample) of 2. (b) False positive variants reported by all tools, with a minimum sample threshold (min_sample) of 2. (c) *F*1 score and true positive rate of all variant calling tools, with a minimum sample threshold (min_sample) of 5. (d) False positive variants reported by all tools, with a minimum sample threshold (min_sample) of 5.

To further validate the performance of MetaQuad, we compared it with several variant calling tools (details are provided in Section 2). We evaluated the performance of these tools using *F*1 scores, true positive rates, and false positives. While MetaQuad was developed to detect informative variants in a group of samples, other tools have their own applicable scenarios. To ensure a more representative comparison of the performance of different tools, we applied filters to the data. For the other tools, we considered only minor variants that appeared multiple times as informative variants, as a counterpart to MetaQuad’s minimum number of samples in the clustering. Additionally, only SNPs were considered in the comparison, and indels were removed during the filtering process. The comparative performance is shown in Fig. 3.

The combined performance of MetaQuad outperformed all the other competitors, especially with higher min-samples. All tools performed better with higher mean depths, with nearly 1 *F*1 scores and almost 100% true positive rates (Fig. 3a and c). In contrast, Varscan2 and MetaSNV could achieve high true positive rates in the case of high mean depth, but at the same time, false positive rates were also significantly increased (Fig. 3b and d). Bcftools and GATK performed well even with low mean depths, but they reported some sequencing errors, and they may miss some informative variants with higher min-samples and low mean depths.

Although MetaQuad may have some false positives in low mean depths, it achieved high true positive rates. However, all the false positive variants can be filtered out if we apply a filter of mean AD and DP of the data (Supplementary Fig. S2). In this case, MetaQuad was better than all the other tools. If many sequencing errors and misalignments are anticipated in the study, MetaQuad will be the ideal method to apply.

In addition to the previous simulation involving a relatively small sample size (*n* = 100), we extended our testing of MetaQuad to a much larger sample size (*n* = 1000). We utilized the same dataset as in the previous simulation, maintaining a mean depth of 11.8, but increased the sample size for each group by 10-fold. Notably, MetaQuad successfully identified 7598/7638 true positive mutations without producing any false positive results, using a minsample threshold of 50 (5% of the total sample size). This robust performance further affirms the applicability of MetaQuad to large-scale datasets.

### 3.3 MetaQuad discovers antibiotic-associated variants in human gut microbiome

**Figure 4.**
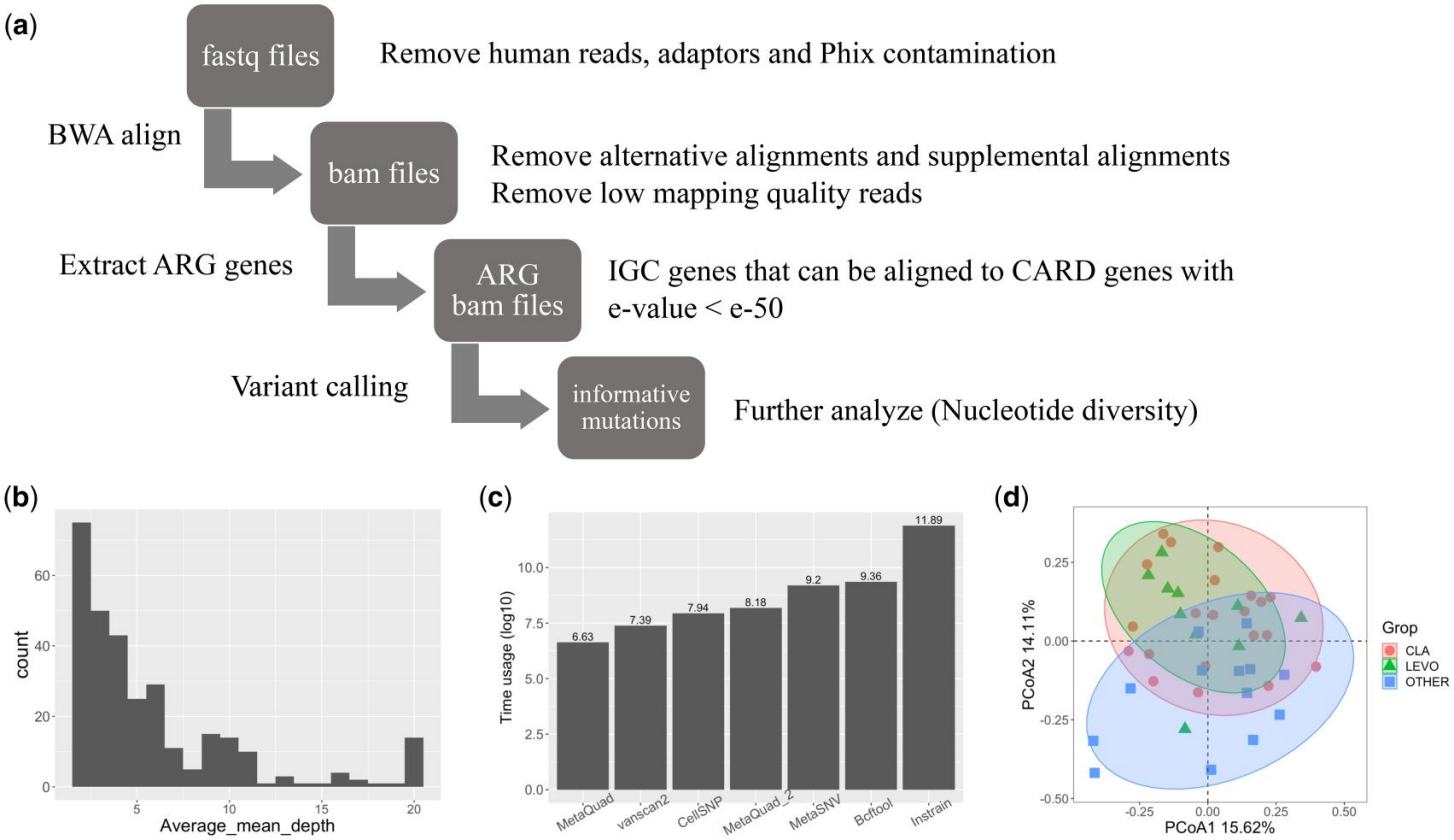
The impact of antibiotics on the human gut microbiome through antibiotic-associated variants. (a) The pipeline used for detecting shared informative variants in the ARG dataset. (b) Average mean depths of each ARG. (c) The time usage of all variant calling tools in the ARG dataset, presented in log 10 transformation. MetaQuad2: total runtime of MetaQuad and cellsnp. (d) PCoA plot of shared informative variant 6 weeks after antibiotic treatment.

MetaQuad was subsequently applied to an ARG dataset after undergoing pre-processing (Section 2 and Fig. 4a) (Wang *et al.* 2023). The samples were divided into three groups based on their clinical treatment: CLA, LEVO, and OTHERS. These samples were obtained from individuals who underwent various antibiotic treatment regimens. Detailed clinical information can be found in the Supplementary Material (ARG_treatment_infor_modified.xlsx). The minSample parameter of MetaQuad was set to 6, representing 5% of the total sample size. To minimize false positive variants, any variants with a mean AD <0.1 and DP <0.5 were removed.

To investigate the impact of antibiotic treatment, we focused solely on ARGs. The mean depths of the ARGs were calculated by samtools coverage and were averaged by the total 121 samples (Fig. 4b). Most ARGs had a mean depth lower than 3. The MetaQuad pipeline demonstrated high efficiency, striking a balance between predictive performance and runtime costs. MetaQuad accurately identified 7591 informative variants from the ARG dataset, showcasing its exceptional efficiency. Notably, it achieved this feat in a mere 60 min, swiftly detecting informative variants from the ARG bam files (Fig. 4c). While vanscan2 emerged as the fastest tool, cellsnp+MetaQuad took slightly longer, with the remaining tools requiring significantly more time to complete the computation. In fact, MetaQuad outperformed vanscan2 in terms of accuracy in our simulated dataset benchmark, further highlighting its superiority.

We analyzed samples from various groups taken at different times, focusing on the allele frequencies of shared informative variants. To analyze the relationship between antibiotic treatment and the allele frequencies of these variants, we used principal coordinate analysis (PCoA) and multilevel pairwise comparison (Fig. 4d). Prior to the analysis, we filtered out variants with NA values and calculated the allele frequencies based on the output of cellsnp-lite (AD/DP). Across all groups, we identified a total of 125, 25, and 40 shared informative variants in the samples before treatment, 6 weeks after treatment (T + 6 W), and 6 months after treatment (T + 6M), respectively. All subsequent analyses were based on these variants. Interestingly, at T + 6 W, the informative variants clearly differentiated the CLA samples and LEVO samples from the others (Fig. 4d). However, this performance was not observed in the samples from the other two time points (Supplementary Fig. S3). Multilevel pairwise comparison revealed that the adjusted *P*-values of LEVO versus OTHER and CLA versus OTHER were significant at T + 6 W, with values of 0.03 and 0.003, respectively. However, none of these comparisons were significant at the other time points. These results may be attributed to natural selection, as the variants emerged following antibiotic treatment.

### 3.4 Shift in nucleotide diversity of antibiotic-resistant genes

We also examined the nucleotide diversity of antibiotic-resistant genes, which typically reflects the accumulation of variants. Informative variants were identified in 529 ARGs, and we filtered out genes with low nucleotide diversity, leaving 15 samples with a nucleotide diversity >0.005. We then focused on the remaining 13 genes to investigate changes in nucleotide diversity before and after antibiotic treatment using the Wilcoxon test. In patients treated with LEVO, the nucleotide diversity of seven genes showed significant differences (*P* < .05) between the 00 and 01 groups (Supplementary Fig. S4). The changes in nucleotide diversity were consistent across all seven genes, with Group 01 showing significantly higher nucleotide diversity than Group 00. Additionally, we observed a relatively reduced nucleotide diversity in Group 02 when compared to Group 01. These findings may help explain how bacteria adapt to antibiotic drugs, where resistance arises due to the accumulation of variants in specific resistance-determining regions (Moon *et al.* 2010). Variants accumulate under antibiotic pressure, but gradually return to pre-perturbation steady states. The new microbial genomic variants detected under antibiotic exposure are only temporary and disappear once the antibiotic pressure is removed.

## 4 Discussion

Detecting variants in shotgun metagenomic data has long been a challenging task due to the presence of common background noise. In this study, we propose and demonstrate the effectiveness of a novel analysis pipeline called MetaQuad for discovering shared informative variants utilizing shotgun metagenomic sequence samples processed by cellsnp-lite.

One of the key strengths of MetaQuad is its unique selection approach in evaluating informative variants, which is more accurate than simply considering the number of samples in which variants were detected. This is particularly important because sequencing depth can vary across samples, making it difficult to consistently detect variants. MetaQuad overcomes this limitation by successfully detecting shared informative variants even with low coverage data.

To evaluate the performance of MetaQuad, we compared it against other common variant calling tools. The results showed that MetaQuad outperformed all these tools, especially for true positive variants with low mean depths. MetaQuad stands out from other tools with its relatively high performance and fast speed. In contrast, the other tools either perform poorly or require too much time to achieve comparable results. This makes MetaQuad a promising tool for various applications in the field.

However, there are some limitations to the MetaQuad pipeline that should be considered. Firstly, it is an alignment-based method, meaning the selection of the reference database can significantly affect the results. Furthermore, the alignment process can be time-consuming and require a substantial amount of RAM space. Another limitation is its dependency on cellsnp-lite and the need to specify genomes or species in the usage of cellsnp. This requirement may make it less convenient to use compared to other tools that do not have such dependencies.

Nevertheless, MetaQuad remains particularly relevant as researchers are increasingly focusing on strain-level differences from high-depth sequencing, making dealing with a large amount of noise inevitable. MetaQuad provides a new way to filter out useless variants and sequencing errors, thus uncovering the untapped potential of information on bacterial variants from sequencing data. Overall, MetaQuad opens up a new paradigm of analysis pipeline for shared informative variant detection in a population of shotgun metagenomic sequencing files. Its ability to detect variants even with low coverage data makes it a valuable tool for researchers working in the field of metagenomics.

## Supplementary Material

vbae030_Supplementary_Data

## Data Availability

All the code for MetaQuad can be found at https://github.com/holab-hku/MetaQuad.
